# Cloud-based introduction to BASH programming for biologists

**DOI:** 10.1093/bib/bbae244

**Published:** 2024-07-23

**Authors:** Owen M Wilkins, Ross Campbell, Zelaikha Yosufzai, Valena Doe, Shannon M Soucy

**Affiliations:** Genomic Data Science Core, Center for Quantitative Biology (COBRE), Dartmouth College, 1 Medical Center Drive, Lebanon, NH 03766, United States; Department of Biomedical Data Science, Geisel School of Medicine, Dartmouth College, 1 Medical Center Drive, Lebanon, NH 03766, United States; Dartmouth Cancer Center, Geisel School of Medicine, Dartmouth Health, 1 Medical Center Drive, Lebanon, NH 03766, United States; National Institutes of Health, 9000 Rockville Pike, Bethesda, MD 20892, United States; Health Data and AI, Deloitte Consulting LLP, 1919 N Lynn St, Suite 1500, Arlington, VA 22209, United States; Google Cloud, 1900 Reston Metro Plaza, Reston, VA 20190, United States; Genomic Data Science Core, Center for Quantitative Biology (COBRE), Dartmouth College, 1 Medical Center Drive, Lebanon, NH 03766, United States; Department of Biomedical Data Science, Geisel School of Medicine, Dartmouth College, 1 Medical Center Drive, Lebanon, NH 03766, United States; Dartmouth Cancer Center, Geisel School of Medicine, Dartmouth Health, 1 Medical Center Drive, Lebanon, NH 03766, United States

**Keywords:** bioinformatics, data Science, training, education, cloud computing

## Abstract

This manuscript describes the development of a resource module that is part of a learning platform named ‘NIGMS Sandbox for Cloud-based Learning’, https://github.com/NIGMS/NIGMS-Sandbox. The overall genesis of the Sandbox is described in the editorial authored by National Institute of General Medical Sciences: NIGMS Sandbox: A Learning Platform toward Democratizing Cloud Computing for Biomedical Research at the beginning of this supplement. This module delivers learning materials introducing the utility of the BASH (Bourne Again Shell) programming language for genomic data analysis in an interactive format that uses appropriate cloud resources for data access and analyses. The next-generation sequencing revolution has generated massive amounts of novel biological data from a multitude of platforms that survey an ever-growing list of genomic modalities. These data require significant downstream computational and statistical analyses to glean meaningful biological insights. However, the skill sets required to generate these data are vastly different from the skills required to analyze these data. Bench scientists that generate next-generation data often lack the training required to perform analysis of these datasets and require support from bioinformatics specialists. Dedicated computational training is required to empower biologists in the area of genomic data analysis, however, learning to efficiently leverage a command line interface is a significant barrier in learning how to leverage common analytical tools. Cloud platforms have the potential to democratize access to the technical tools and computational resources necessary to work with modern sequencing data, providing an effective framework for bioinformatics education. This module aims to provide an interactive platform that slowly builds technical skills and knowledge needed to interact with genomics data on the command line in the Cloud. The sandbox format of this module enables users to move through the material at their own pace and test their grasp of the material with knowledge self-checks before building on that material in the next sub-module.

This manuscript describes the development of a resource module that is part of a learning platform named ``NIGMS Sandbox for Cloud-based Learning'' https://github.com/NIGMS/NIGMS-Sandbox. The overall genesis of the Sandbox is described in the editorial NIGMS Sandbox [1] at the beginning of this Supplement. This module delivers learning materials on the analysis of bulk and single-cell ATAC-seq data in an interactive format that uses appropriate cloud resources for data access and analyses.

## Introduction

The next-generation sequencing (NGS) revolution has generated massive amounts of new data and transformed the field of genomics. Furthermore, development of new and existing genomics technologies continues to increase throughput of existing platforms while also giving rise to novel data types that measure an ever-growing list of genomic modalities [2, 3]. Bioinformatic, computational and statistical analysis approaches are critical for extraction of meaningful biological insights from the highly dimensional datasets generated by NGS technologies. Application of complex bioinformatics approaches has played a central role in recent scientific milestones, such as the completion and closure of the entire human genome sequence [4], and rapid assembly of the Sars-CoV-2 genome during the COVID-19 pandemic [5]. As throughput of existing genomics platforms have increased; labs routinely utilize data generated by high-throughput sequencing technologies in their research projects, increasing demand for scientists able to extract meaningful biological insights from these data. Analyzing these data requires skills from biology, statistics and computer science. Many biologists are experts in their specific biological domain but have less familiarity with statistical approaches and computer science. This creates a bottleneck where scientists are becoming increasingly reliant on a small number of research labs and core facility scientists with the expertise to analyze NGS data. The disconnect between those who are designing the experiments and conducting downstream analyses can have detrimental effects on quality of the data produced. Improved understanding of analytical methodology enhances the ability of experimental researchers to plan experiments, interpret biological insights from their data and explore their own datasets without the supervision of a trained bioinformatician.

Though the field is growing at a rapid pace, and biology students trained in data science have more employment opportunities available to them, many life sciences students earn their degree with little exposure to bioinformatics [6]. A 2019 survey of life sciences faculty from primarily undergraduate institutions across the United States attempted to understand barriers to integrating data science topics into biology curricula [6]. The survey reported that a lack of expertise in faculty, a lack of basic computing knowledge in students, outdated equipment and limited access to compute resources were seen as major barriers to incorporating bioinformatics education to life sciences curriculum at these institutions [6]. Other challenges reported include access to appropriate software, difficulty communicating computational processes in a biological context and rapid changes in analytic techniques and technology [6]. These challenges are representative of those faced by IDeA states. The Fundamentals of Bioinformatics module developed here attempts to mitigate some of these challenges by demystifying access to state-of-the-art computer technology available through cloud platforms and creating an opportunity to develop a strong foundation for working with biological data through a terminal interface.

The Fundamentals of Bioinformatics module is meant for scientists with no previous programming experience to familiarize themselves a command line environment through the Google Cloud infrastructure, while building the proficiency necessary to work with modern biological datasets. As NGS data structures become more complex, there is a growing fascination with flashy computational approaches leveraging artificial intelligence or neural networks; however, the majority of genomic data science relies on a strong foundation of basic BASH coding operations covered in this module. This module was designed to bridge gaps in knowledge observed in hundreds of students in a multitude of bioinformatics training programs. Namely, these gaps are (1) the basic components of compute infrastructure, (2) basic programming proficiency and (3) software management. Absence of these three fundamental skills complicates training of more advanced topics as students lack the fundamental skills required to interface with datasets and leverage analytical tools. Trainees who complete this module will build experience with BASH programming within a cloud compute environment, and learn to leverage compute resources efficiently, regardless of the resources available at their local institution. The module begins with an overview of cloud compute infrastructure and an introduction to working with a general terminal interface (referred to henceforth simply as the ‘terminal’). Next, the module introduces the format of, and information contained in, standard genomic file formats along with basic commands for interacting with them in the terminal. Lastly the module explains best practices for installing and managing software using package managers. The module culminates with an exercise requiring trainees to create a software environment and design terminal commands for each step in a genome assembly workflow. Sub-modules build in complexity throughout the lesson and utilizes self-checks to test that trainees understand the material before moving forward in the lesson.

## Method and implementation

This module is designed to introduce basic foundations for interacting with NGS data on the command line using Google Cloud. The assumption is that trainees have never used the terminal interface and have no experience with programming, cloud computing or software installation. The design of these ‘sandbox modules’ enables users to move through lessons at their own pace and ‘play’ with the tools in a contained environment without incurring large costs. Each lesson is presented in the format of a Jupyter notebook, and the majority of code within the notebook can be run from within the notebook. However, completing a complex biological analysis in the scope of a Jupyter notebook is not feasible. In the first lesson, trainees are instructed to open a terminal window linked to the cloud compute instance used for the lesson. In subsequent lessons, trainees are encouraged to copy the code from the notebook and execute the commands in the terminal window. Ideally, trainees will begin by ‘playing’(editing) with the code within the Jupyter notebooks, then move on to editing the same code in the linked terminal interface and finally attempt to edit the code such that it would be applied to their own full-sized datasets using the terminal. Throughout the module, each new concept in the submodules ends with a TEST YOUR SKILLS section where trainees will complete coding challenges that leverage their cumulative knowledge throughout the module. These sections become more difficult as trainees move through the module. Each TEST YOUR SKILLS block is followed by a flashcard; one click on the flashcard will reveal a hint, and a second click will reveal the answer to the TEST YOUR SKILLS code block.

### Submodule 1: introduction to terminal

This lesson will prepare trainees to work with data in subsequent lessons by introducing the two interfaces used in all modules: the Jupyter notebook and the terminal interface. The Jupyter notebook contains the lesson material, practice code chunks and knowledge self-checks. This format enables trainees to customize a copy of the notebook by taking notes, testing code and recording results directly within the notebook. This lesson further explores the advantages of learning to use the terminal interface and the basic syntax of BASH commands. At the end of the submodule, trainees test their skills by first writing a series of BASH commands to list files in a directory in various formats and then creating several directories and navigating through the directories with absolute and relative paths.

### Submodule 2: introduction to cloud computing

This lesson introduces the concepts behind cloud computing. We describe the components of a virtual machine and the benefits of parallelizing large computing tasks, like read mapping or variant calling. Trainees will be introduced to Google buckets and pull some data from the cloud with the gsutil (v5.24) tool suite; these data will be used in submodules 3–5.

### Submodule 3: genomics file formats

This submodule introduces three commonly used NGS file formats for storing genomic data: the FASTA format, which stores basic sequence data, FASTQ format, which stores raw read sequences, and GFF format, which stores genome annotation data. We discuss how to access data from each of these file types. This submodule builds on BASH proficiency learned in submodule 1 with additional BASH commands like `*grep*` and the pipe operator (|) to build more complex code. The TEST YOUR SKILLS sections in this lesson ask users to manipulate the information returned from a command with different combinations of arguments to get general information about files and answer specific queries about the data (e.g. how many base pairs in the first 10 000 records of a FASTQ file have a Q-score of 20).

### Submodule 4: beyond basic BASH

This submodule begins by reviewing BASH commands from previous lessons and then introduces looping and scripting. Given that the power in writing code is performing a repetitive task, in this lesson, we extend on the material from the previous lesson to run loops over complex code chunks for multiple files with a single command. We then demonstrate how code can be aggregated into a script that can be applied to future datasets. Trainees are challenged to write a loop that returns a series of numbers and test how the output of a loop changes when using the redirect (>) and the append (>>) commands.

### Submodule 5: installing and managing bioinformatics software

In this submodule, trainees will learn how to customize a compute environment by installing a suite of tools to perform a specific analysis using the conda package manager. Writing bioinformatics analysis pipelines requires chaining together specialized analysis tools where the output of one tool is the input of the next tool. These tools are usually developed by the open-source community in isolation of each other and can have huge numbers of specific software dependencies. Use of package management systems such as conda has become critical to ensure software compatibility through creation of discrete software environments, which contain a collection of software with specific versions that can be different to those installed on your local computer. This submodule leverages *fastQC* (v0.11.8) [7] and *multiQC* (v1.6) [8] to demonstrate how to build conda environments. Trainees are required to enable conda to create a kernel on their virtual machines (VMs), create and modify a conda software environment and check the quality of FASTQ files with a loop.

### Submodule 6: putting it all together

This submodule combines the skill sets of previous submodules to run a genome assembly workflow. Trainees create two conda environments, demonstrating the utility of multiple environments to use software that leverage conflicting dependencies. Trainees use the *sra-toolkit* (v2.5.7) to download data from NCBI’s Sequence Read Archive (SRA), *fastqc* (v.0.11.8) [7] to check raw data quality, *spades* (v3.15.5) [9] for genome assembly, *prokka* (v1.14.6) [10] for gene annotation and *gsutils* (v5.24) to write the final file set to a Google bucket. Trainees are provided with limited code in this submodule and must author commands for the quality control, genome annotation and to write data to a new Google bucket. Following completion of this submodule, trainees will be able to run a basic genome annotation pipeline in a cloud environment and will have learnt transferable skills for types of bioinformatics analysis.

### Submodule 7: error mitigation

Learning how to understand and address errors produced in a typical bioinformatics pipeline is an essential skill for computational analysis of biological datasets. This lesson will help trainees learn to interpret and mitigate common errors encountered when working in the terminal environment or creating a conda environment. The TEST YOUR SKILLS section in this lesson involves debugging broken code.

## Results

**Figure 1:**
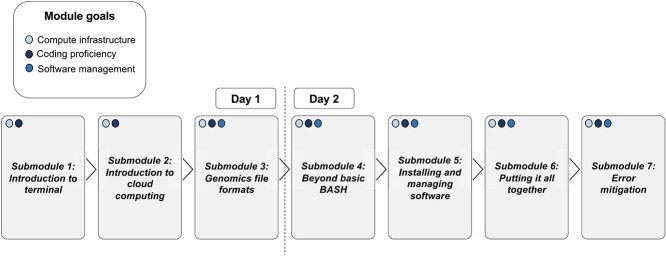
Module schematic. This figure shows the flow of submodules that make up the Fundamentals of Bioinformatics module. The goals of the module are delineated in the key on the top left of the figure; the number and color of dots in the upper-left corner of each module rectangle indicate the goals met by the submodule. For beginners, we advise breaking the module up into two parts to permit time to ‘play’ with the commands in each module.

The Fundamentals of Bioinformatics module provides basic instructions for working on the terminal interface with NGS files, installing bioinformatic software and setting up an NGS analysis. The instructions for each of these steps are generalizable to most analytic workflows and importantly will build ‘muscle memory’ for working with data on the command line, which is often a barrier to trainees working with NGS data. The goal of this module is to build familiarity with the components of compute infrastructure (submodules 1–2), to gain experience working within the terminal environment on the cloud (submodules 1–4) and to install and utilize bioinformatics software to run an analytic workflow (submodules 5–6) (Fig. 1). The module begins by explaining the advantages of using the terminal interface over point and click graphical user interfaces (GUIs) (submodule 1) and how to select appropriate compute resources for an analysis project (submodule 2). Submodules 3 and 4 build a foundation of BASH coding skills, while trainees work with common NGS file formats (FASTA, FASTQ and GFF) within the terminal interface. Submodule 5 introduces best practices for software installation with the conda software package manager. In submodule 6, trainees create a conda environment for a genome assembly project and use software manuals to build an analysis workflow and write a final file set to a Google bucket. Lastly, submodule 7 provides a framework for how to respond to errors that arise when analyzing data on the terminal.

This module uses example data from several diverse sources. These files were intentionally selected to come from diverse sources to accentuate the flexible application of the foundational skill sets contained in this module. Submodule 2 further accentuates this point by outlining the range of analytic time required for multiple bioinformatic analyses depending on the compute resources requested. In submodule 3, a FASTA file and GFF file for the severe acute respiratory syndrome coronavirus 2 (SARS CoV2) isolate Wuhan-Hu-1 [5] is used as well as a FASTA file containing the S11 ribosomal protein sequences from several proteobacteria [11]. FASTQ files used in submodules 3 and 4 are down-sampled from transcriptomic data of human airway smooth muscle cell lines treated with various glucocorticoids [12]. These data were down-sampled to include only reads that map to chromosome 20. Lastly, FASTQ files in submodule 6 are part of a surveillance effort for SARS CoV2 infections in western New Hampshire. Files analyzed in this module were selected to minimize compute requirements and thus the resources needed to run this module; in doing so, users are encouraged to take sufficient time ‘playing’ with the code in the terminal to transition from understanding what a command is doing to understanding how to write your own command.

## Discussion and conclusion

Optimization of data analysis ecosystems is an important area of investment for bioinformatic sciences. Biomedical research is increasingly utilizing high-dimensional genomics data (single-cell and spatial ‘omics), and many smaller institutions struggle to maintain onsite infrastructure to process and store these files [6]. Long-term data storage is a challenge, even for institutions that are well resourced, due to the increasing size and complexity of the data. Furthermore, data analyses are increasingly complex, which taxes local computing resources and is often a rate-limiting step for deploying analysis workflows. Lastly, collaboration between groups at different institutions often requires sharing of data and analytic pipelines; however, these pipelines need to be reconfigured to work within the compute infrastructure of each institution. Data analytics that leverage cloud compute environments provide access to the latest technology and centralize the cost and labor of investing in and maintaining this technology, ultimately leading to cost savings for all cloud users who might otherwise need to invest in these systems or are limited to analyses that leverage older technology. Cloud compute systems utilize multiple storage tiers enabling users to make use of low-cost cold storage for data that are not frequently accessed; this significantly reduces the cost of storing raw data files in particular. Analysis workflows that are designed to run on the cloud can be deployed by anyone with access to them without needing to redesign the workflow; this promotes inter-institutional collaboration. The benefits of leveraging cloud compute resources for data science are only relevant if the cloud is accessible to data scientists. Two major barriers to scientists that could benefit from utilizing cloud computing are a lack of basic programming skills and technology adoption. The development of cloud-based data science training modules will enable researchers to build data analytics skill sets within the cloud environment rather than trying to learn within one system and then adapt those skills to another system.

This module in particular addresses both the barrier of technology adoption and the lack of coding skills concurrently by slowly introducing basic programming skills. Unlike much of the material life science students are exposed to, programming is a skill that is learned through processes analogous to ‘muscle memory’ and this ‘sandbox’ format is ideal for building muscle memory through ‘playing’ with the coding examples in each submodule. Furthermore, the material in this module consists of fundamental programming knowledge and will be relevant even as analytic techniques continue to evolve at a rapid pace. For example, though we fully expect that best practices for software installation will move toward more fully containerized environments (singularity and docker), the basic premise of a software container can be learned by working with conda environments and will endure even as best practices evolve. Building a strong computational foundation for working with biological data will enable trainees to take better advantage of freely available tutorials and remain up to date with the latest analytic techniques for working with new NGS data types.

This project had several components that were managed effectively through collaboration between the private sector (Google and Deloitte), academic sector (Dartmouth College) and federal agencies (NIGMS and New Hampshire IDeA Networks of Biomedical Research Excellence (NH-INBRE)). This team approach to building this resource was instrumental to ensure that the module was completed on time, was synergistic with other modules being developed, efficiently leveraged cloud computing technology and effectively addressed knowledge gaps to build a strong foundation for working in the cloud. This project benefited strongly from the cloud expertise of team members from the private sector; this significantly expedited the development and design of lessons such that they were in line with best practices for working in the cloud. One caveat of these lessons is that trainees will have access to documentation only and will not have the benefit of an instructor to guide them through the material. A lack of interaction is one of the major reasons cited for the high attrition rate of massively online open courses, which some studies estimate is as high as 95% [13]. One way to mitigate this issue is to utilize these training modules to complement existing training programs; for example, pairing this module with bioinformatics workshops could provide students with a place to build experience with BASH programming prior to the workshop so during the course, they can focus on the algorithmic and statistical concepts. This would expose undergraduate life science students to foundations of biological data science and enhance their employability and preparedness for further education [6]. Though all lesson materials are available through the NIGMS github page, utilizing these lessons requires users to set up and purchase compute resources through the Google Cloud Platform. The cost of running these analyses is unlikely to be prohibitive (this module costs $2 to run); however, the expertise required to set up a virtual machine using the documentation alone may be a barrier to their utilization (even with documentation available from National Institutes of Health, https://training.nih-cfde.org/en/latest/Cloud-Platforms/Introduction-to-GCP/gcp2/). Currently, access to these modules in IDeA states is supported through NIGMS, which funds access for a limited number of students annually who sign up through their local INBRE group. This mechanism for controlling access provides IDeA state users with access to an individual within their local INBRE network that has experience to guide users through the setup process.

**Key Points**

- Data analyses are increasingly complex, and there is a paucity of introductory computational training mechanisms for bench scientists.
- Establishing foundational skills for working with computational data promotes a better understanding of complex algorithms applied to genomic data. This understanding leads to improved experimental plans and the generation of higher-quality scientific datasets.
- Cloud computing has the potential to democratize complex data analytics when it is paired with widely accessible training programs aimed at building skills needed to leverage cloud compute resources (i.e. basic BASH programming).

## Data Availability

[Wuhan-Hu-1 complete genome sequence and annotation files]^*^ Wu F., Zhao S., Yu B., *et al.*, 2020 [5], Severe acute respiratory syndrome coronavirus 2 isolate Wuhan-Hu-1, complete genome, GenBank, NC_045512.2 [S11 ribosomal sequences]^*^ Shakya M., Soucy S.M., Zhaxybayeva O., 2017 [11] [Downsampled RNAseq data from airway cell lines]^*^ Himes B.E., Jiang X., Wagner P., *et al.* 2014 [12], Sequence Read Archive, SRP033351 [Raw whole genome sequence data for Sars Co-V2]^*^ Sequence Read Archive, SRR18435413 All codes are available at GitHub (https://github.com/NIGMS/Fundamentals-of-Bioinformatics).

## Notes

Drs **Shannon Soucy** and **Owen Wilkins** co-direct Dartmouth College’s Genomic Data Science Core (GDSC), a core facility within the Center for Quantitative Biology. GDSC specializes in advanced genomic data analysis and develops training tools for cutting-edge analytic methods.
